# CD34 and HLA‐DR Double Positive Microgranular Acute Promyelocytic Leukaemia With Multiple T‐ and B‐Cell Markers Expression

**DOI:** 10.1002/jha2.70106

**Published:** 2025-08-22

**Authors:** Ke Xu, Evan Vitsaras, Rajeev Gupta

**Affiliations:** ^1^ Department of Haematology University College London Hospitals NHS Foundation Trust University College London London UK; ^2^ Specialist Integrated Haematology Malignancy Diagnostic Service Health Services Laboratories University College London Hospitals NHS Foundation Trust London UK

1

A 39‐year‐old male presented with fatigue. Bloodwork showed haemoglobin 53 g/L, white blood cell 2.3 × 10^9^/L, platelets 24 × 10^9^/L, and slightly deranged clotting screen (fibrinogen 2.7 g/L, PT 15 s, APTT 30 s). A bone marrow aspirate was effaced by blasts with blebbed basophilic cytoplasm and occasional Auer rods (Figure 1A). Multi‐parameter flow cytometry showed the blasts were positive for CD34, HLA‐DR, CD117, CD33, CD13, CD19, CD2, CD5, CD7, CD56, and myeloperoxidase, and negative for cCD3 and terminal deoxynucleotidyl transferase (Figure 1B). About 20% of blasts were CD38 negative, suggesting immaturity, which predicted adverse genetic risk. However, rapid combined exome/RNA sequencing (Oncomine Myeloid Assay GX v2) identified a *PML::RARA* bcr1 fusion, confirmed by fluorescence in situ hybridisation, with no other abnormalities. Acute promyelocytic leukaemia (APL) was diagnosed. With low white cell count and mild coagulopathy, this case was classified as standard risk APL. The patient received all‐trans retinoic acid (ATRA) and arsenic trioxide with an uncomplicated course to complete molecular remission.

**FIGURE 1:**
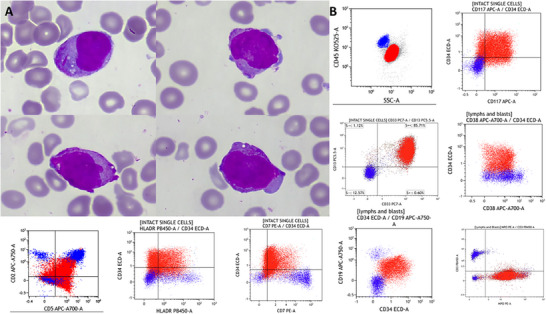
(A) Bone marrow aspirate (May‐Grünwald‐Giemsa stain ×100 objective). (B) Immunophenotyping (red colour population).

APL is a medical emergency. Microgranular APL can display atypical morphology and immunophenotype, which makes the diagnosis challenging [1]. This patient's immunophenotype is highly atypical but does not meet World Health Organization criteria for mixed‐phenotype acute leukaemia [1]. ATRA is normally given to patients with suspected APL ahead of formal molecular diagnosis. Because of the atypical morphology and CD34+HLADR+ surface phenotype, APL was not initially suspected in this case. We recommend routine rapid testing for common fusions in newly diagnosed acute leukaemia to ensure correct classification and timely treatment.

## Author Contributions

K.X. wrote up the manuscript. E.V. and R.G. performed flow cytometry analysis. K.X., E.V., and R.G. critically revised the final version of the manuscript.

## Conflicts of Interest

The authors declare no conflicts of interest.

## Ethics Statement

This article does not contain any studies with human participants performed by any of the authors.

## Clinical Trial Registration

The authors have confirmed clinical trial registration is not needed for this submission.

## Data Availability

The data that support the findings of this study are available from the corresponding author upon reasonable request.
